# EMT and Stem Cell-Like Properties Associated with HIF-2α Are Involved in Arsenite-Induced Transformation of Human Bronchial Epithelial Cells

**DOI:** 10.1371/journal.pone.0037765

**Published:** 2012-05-25

**Authors:** Yuan Xu, Yuan Li, Ying Pang, Min Ling, Lu Shen, Xiaojun Yang, Jianping Zhang, Jianwei Zhou, Xinru Wang, Qizhan Liu

**Affiliations:** 1 Institute of Toxicology, Nanjing Medical University, Nanjing, People's Republic of China; 2 The Key Laboratory of Modern Toxicology, Ministry of Education, School of Public Health, Nanjing Medical University, Nanjing, People's Republic of China; 3 Department of General Surgery, The Second Affiliated Hospital, Nanjing Medical University, Nanjing, People's Republic of China; University of Nebraska Medical Center, United States of America

## Abstract

**Background:**

Arsenic is well-established as a human carcinogen, but the molecular mechanisms leading to arsenic-induced carcinogenesis are complex and elusive. It is not been determined if the epithelial-mesenchymal transition (EMT) and stem cell-like properties contribute in causing to carcinogen-induced malignant transformation and subsequent tumor formation.

**Methods:**

To investigate the molecular mechanisms underlying EMT and the emergence of cancer stem cell-like properties during neoplastic transformation of human bronchial epithelial (HBE) cells induced by chronic exposure to arsenite. HBE cells were continuously exposed to arsenite. Spheroid formation assays and analyses of side populations (SPs) were performed to confirm that arsenite induces the acquired EMT and cancer stem cell-like phenotype. Treated HBE cells were molecularly characterized by RT-PCR, Western blots, immunofluorescence, Southwestern assays, reporter assays, and chromatin immunoprecipitation.

**Results:**

With chronic exposure to arsenite, HBE cells undergo an EMT and then acquire a malignant cancer stem cell-like phenotype. Twist1 and Bmi1 are involved in arsenite-induced EMT. The process is directly regulated by HIF-2α. The self-renewal genes, *Oct4*, *Bmi1*, and *ALDH1*, are necessary for arsenite-mediated maintenance of stem cells.

**Conclusions:**

EMT, regulated by HIF-2α, and the development of a cancer stem cell-like phenotype are associated with arsenite-induced transformation of HBE cells.

## Introduction

The epithelial-mesenchymal transition (EMT) is a developmental process by which epithelial cells are converted to mesenchymal cells during embryogenesis [1]. EMT, which involves loss of cell polarity, decreases in cell-to-cell adhesion, and increased capacity for migration, is necessary for tumor metastasis and organ fibrosis [2]. EMT, however, has not been considered to be involved in transformation of normal cells to malignant cells in the initiation of tumorigenesis [3].

A concept recently proposed to explain the characteristics of neoplastic tissues is the existence of self-renewing, stem-like cells within tumors, which have been called cancer stem cells (CSCs) [4]. Within a tumor, CSCs, which constitute a small portion of neoplastic cells, are defined by their capacity to produce new tumors. For this reason, they have also been termed ‘tumor-initiating cells’ [5]. The process of EMT generates cells with stem-like properties [6]. The link between EMT and induction of cancer stem cells may explain why EMT induces tumor initiation and progression.

Arsenic is well-established as a human carcinogen [7]. A positive correlation exists between arsenic exposure and increased incidences of various forms of cancer, as documented by reports from arsenic-endemic areas of the world [8]. To deal with this problem, it is essential to elucidate molecular mechanism involved in arsenic-induced carcinogenesis.

Exposure to arsenic disrupts the dynamics of stem cells (SCs) in human and rodent skin *in vivo* and *in vitro*, resulting in an overabundance of stem cells/CSCs [9], [10], an event likely involved in development of skin cancer. Arsenite apparently transforms prostate epithelial stem/progenitor cells into cancer stem-like cells that drive oncogenesis [11]. Arsenic affects human stem cells by blocking differentiation pathways, which are considered to be targets in carcinogenesis [12], [13]. Chronic exposure to arsenite triggers expression of the EMT-inducing transcription factors, ZEB1 and ZEB2, resulting in EMT and malignant transformation [14]. The induction of EMT is associated with acquisition of stem cell-like features during malignant transformation induced by other carcinogens [3]. It has not been determined, however, if, in human cells, EMT and stem cell-like properties contributes in causing to arsenite-induced malignant transformation and subsequent tumor formation.

In this study, we investigated the effect of chronic arsenic exposure on the induction of EMT and the acquisition of stem cell-like properties in human bronchial epithelial (HBE) cells. The goal was to determine if arsenite-induced induction of EMT and acquisition of stem cell-like properties are mechanisms associated with arsenic-induced carcinogenesis. We report, for the first time, a link between arsenite exposure, induction of EMT, and the development of a stem cell-like phenotype that together may be associated with malignant transformation and tumor formation. Such information contributes to an understanding of lung oncogenesis caused by arsenite.

## Results

### Chronic arsenite exposure causes EMT of HBE cells

**Figure 1:**
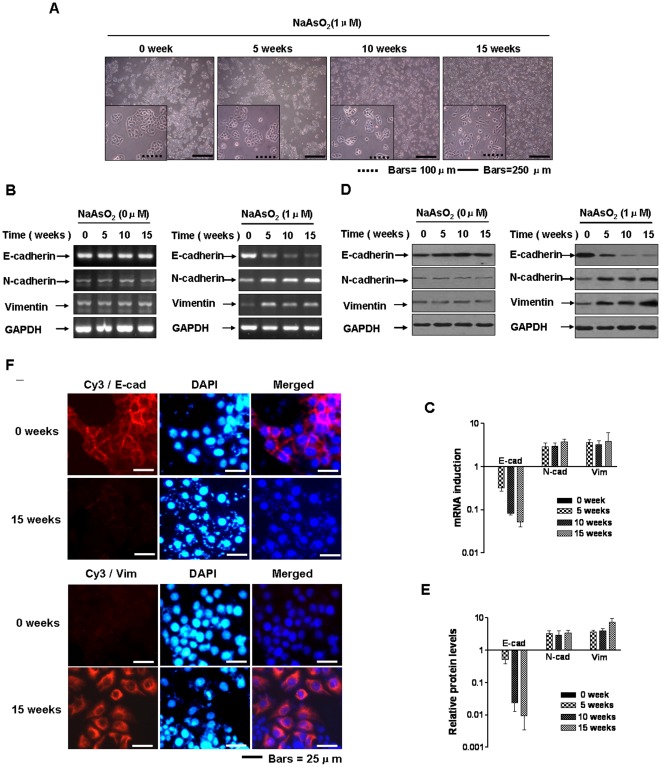
Chronic exposure to arsenite induces EMT in HBE cells. Abbreviations: E-cad, E-cadherin; N-cad, N-cadherin; Vim, vimentin. Densities of bands were quantified by Eagle Eye II software. GAPDH levels, measured in parallel, served as controls. HBE cells were exposed to 0.0 or 1.0 µM arsenite for 0, 5, 10 or 15 weeks. (**A**) Morphology of HBE cells during arsenite treatment for 0, 5, 10, and 15 weeks; bars = 250 µm, or bars = 100 µm. The mRNA levels of *E-cadherin*, *N-cadherin*, and *vimentin* were determined by RT-PCR (**B**) and by quantitative RT-PCR (**C**, means ± SD, n = 3) after HBE cells were exposed to 0.0 or 1.0 µM arsenite for 0, 5, 10 or 15 weeks. ^*^ *P*<0.05 difference from control HBE cells. Western blots (**D**) and relative protein levels (**E**, means ± SD, n = 3) of E-cadherin, N-cadherin, and vimentin in HBE cells exposed to arsenite for 0, 5, 10, or 15 weeks. (**F**) Immuofluorescence staining of E-cadherin and vimentin in HBE cells for the indicated times. Red represents E-cadherin and vimentin staining. Blue represents nuclear DNA staining by DAPI; bars = 25 µm.

A low concentration (1.0 µM) of arsenite increased the neoplastic transformation of HBE cells, as determined by anchorage-independent growth in soft agar and tumorigenesis in nude mice (Figure S1). For HBE cells, alteration from epithelial to spindle-like mesenchymal morphology is a manifestation of arsenite-induced transformation. This change indicates that chronic arsenite exposure causes EMT of HBE cells. To test the hypothesis, HBE cells were exposed to 0.0 or 1.0 µM arsenite for 15 weeks. The alterations from epithelial to spindle-like mesenchymal morphology started at 10 weeks of arsenite exposure and increased thereafter; the cells acquired a fibroblast-like mesenchymal appearance consistent with EMT with increased time of exposure (Figure 1A).

The expression of the EMT markers, E-cadherin, N-cadherin, and vimentin, was determined [15]. After 5 weeks of arsenite exposure, expression of the epithelial marker, E-cadherin, decreased. In contrast, expression of the mesenchymal marker, vimentin, increased with longer times of arsenite exposure (Figures 1B, 1C, 1D and 1E). To determine if the molecular alterations of EMT occurred in control and transformed HBE cells, staining of E-cadherin and vimentin, measured by immunofluorescence microscopy, confirmed the EMT-associated shift in the localization of markers. The transformed cells formed epithelial-like intercellular junctions and displayed increased expression of fibroblast markers (Figure 1D). Hence, both morphological and molecular changes demonstrated that, with chronic exposure to arsenite, HBE cells underwent an EMT.

### Twist1 is involved in arsenite-induced EMT of HBE cells

**Figure 2:**
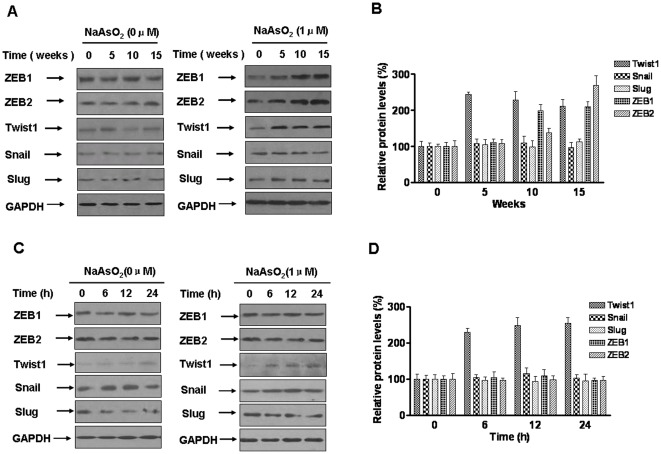
Twist1 is involved in arsenite-induced EMT in HBE cells. Densities of bands were quantified by Eagle Eye II software. GAPDH levels, measured in parallel, served as controls. HBE cells were exposed to 0.0 or 1.0 µM arsenite for 5, 10 or 15 weeks. Western blots (**A**) and relative protein levels (**B**, means ± SD, n = 3) of ZEB1, ZEB2, Twist1, Snail, and Slug were determined in control and treated HBE cells at the indicated times. Western blots (**C**) were performed and relative protein levels (**D**, means ± SD, n = 3) of ZEB1, ZEB2, Twist1, Snail and Slug were measured after HBE cells were exposed to 0.0 or 1.0 µM arsenite for 0, 6, 12, or 24 h.

The process of EMT is controlled by transcriptional factors, including the zinc finger proteins, Snail, Slug, ZEB1, and ZEB2/SIP1, and the basic helix-loop-helix factor, Twist1 [16]. The EMT regulators, ZEB1 and ZEB2, are active in cells chronically exposed to arsenite [14]. The expressions of ZEB1, ZEB2, Snail1, Slug, and Twist1 in control and arsenite-transformed HBE cells were determined. Expression of Twist1 increased with longer times of arsenite exposure, and ZEB1 and ZEB2 expressions were increased starting from about 10 weeks of chronic arsenite exposure (Figures 2A and 2B). To determine if arsenite induces ZEB1, ZEB2, and Twist1 expressions, HBE cells were treated with 0.0 or 1.0 µM of arsenite for 0, 6, 12, or 24 h. In arsenite-treated HBE cells, only the levels of Twist1 increased. ZEB1 and ZEB2 expressions were not changed, and the levels of Snail and Slug were not appreciably altered (Figures 2C and 2D). These results suggest that up-regulation of Twist1 is associated with arsenite-induced EMT.

### HBE cells acquire stem cell-like properties during arsenite-induced EMT

**Figure 3:**
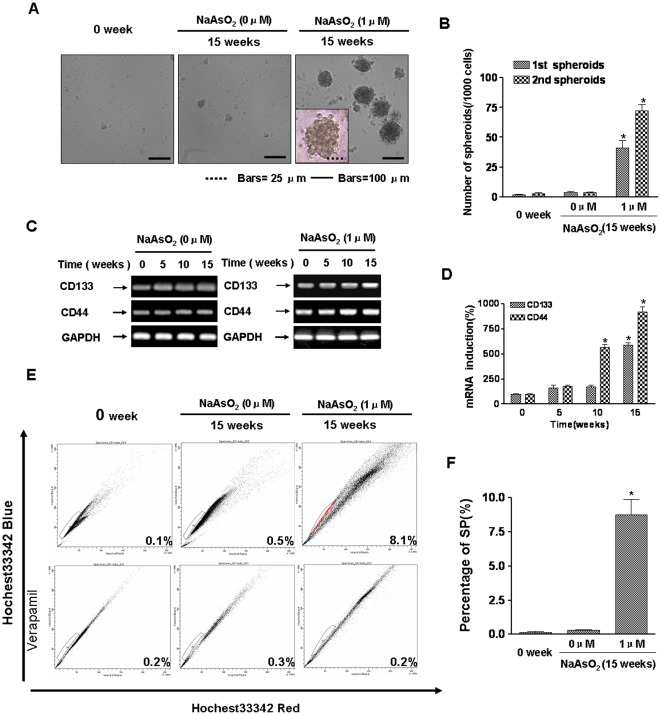
Arsenite-induced EMT of HBE cells causes them to acquire stem cell–like properties. HBE cells were exposed to 0.0 or 1.0 µM arsenite for 15 weeks. (**A**) Phase-contrast images of the primary spheroids that were seeded by control HBE cells, untreated cells, and cells treated with arsenite for 15 weeks. (**B**) The primary spheroids were dissociated into single cells and cultured for secondary spheroids; the primary and secondary spheroids formed were quantified (means ± SD, n = 3); bars = 25 µm, or bars = 100 µm, ^*^ *P*<0.05 difference from control cells. The mRNA level of *CD44* and *CD133* were determined by RT-PCR (**C**) and by quantitative RT-PCR (**D**, means ± SD, n = 3) after HBE cells were exposed to 0.0 or 1.0 µM arsenite for 0, 5, 10 or 15 weeks. ^*^ *P*<0.05 difference from control HBE cells. (**E**) Control cells, untreated cells, and HBE cells treated with arsenite for 15 weeks were fixed, and SP cells were analyzed by FACS. (**F**) The percentages of SP cells in the gated area are shown for cells. ^*^ *P*<0.05 different from control HBE cells.

Since induction of EMT has been associated with the acquisition of stem cell-like features, including nonadherent growth and changes in expression of cell-surface glycoproteins [17], the capacity of HBE cells for formation of spheroids during arsenite-induced EMT was determined. Formation of spheroids demonstrates the capacity of cells for self-renewal and for initiation of tumors [18], which are characteristics of stem cells. In arsenite-induced EMT of HBE cells, there was an increase in formation of spheroids (Figure 3A).

To test the self-renewal capacity of the sphere-forming cells, the primary spheroids were dissociated into single cells, and secondary spheroid assays were performed [18]. The number of secondary spheroids was greater than for primary spheroids (Figure 3B). CD133 and CD44 are cell-surface markers of lung stem cells [19], [20]. During the arsenite-induced EMT of HBE cells, there were increased levels of mRNAs for *CD133* and *CD44* (Figures 3C and 3D). SP cells, which are enriched along with stem cells, provide an alternative source for markers that is particularly useful in situations where molecular markers for stem cells are unknown [21]. Flow cytometric analysis indicated that the percentage of SP cells increased in the arsenite-induced EMT of HBE cells (Figure 3E). These data demonstrate that HBE cells acquire stem cell-like characteristics by chronic exposure to arsenite.

### Self-renewal genes are over-expressed during arsenite-induced acquisition of the stem cells-like phenotype

**Figure 4:**
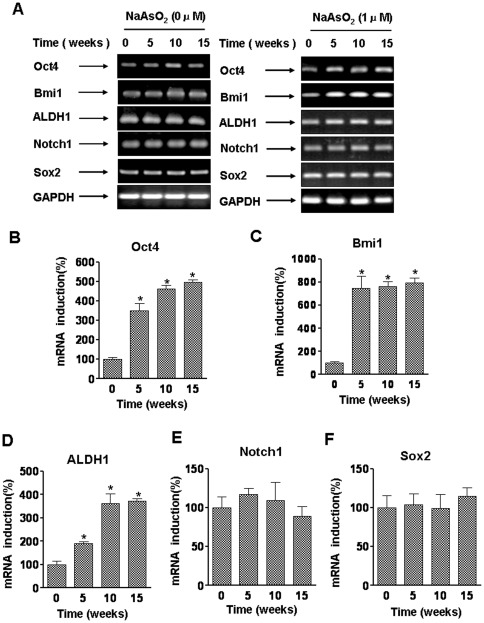
Oct4, Bmi1, and ALDH1 are over-expressed during arsenite-induced acquisition of the stem cell-like phenotype. HBE cells were exposed to 0.0 or 1.0 µM arsenite for 5, 10, or 15 weeks. (**A**) The mRNA levels of *Oct4, Bmi1, ALDH1, Notch1,* and *Sox2* were determined by RT-PCR. Quantitative RT-PCR (means ± SD, n = 3) was used to measure the transcript level of *Oct4* (**B**), *Bmi1* (**C**), *ALDH1* (**D**), *Notch1* (**E**), and *Sox2* (**F**) after HBE cells were exposed to 0.0 or 1.0 µM arsenite for the indicated times. ^*^ *P*<0.05 difference from control cells.

The expression of self-renewal genes during arsenite-induced acquisition of the stem-cell like phenotype was examined. In CSCs from various cancers, there is expression of the key ‘stemness’ genes, *Oct-4*, *Bmi1*, *Notch1*, *ALDH1*, and *Sox2* [22], [23], [24]. As determined in the present study, with longer time of exposure to arsenite, there was increased expression of mRNAs for *Oct4, Bmi1,* and *ALDH1*; however, there were no substantial changes in expressions of mRNAs for *Notch* and *Sox2* (Figures 4A–4E). These results indicate that the self-renewal genes, *Oct4*, *Bmi1*, and *ALDH1* are necessary for arsenite-mediated maintenance of stem cells.

### Bmi1 is involved in arsenite-induced acquisition of stem cell-like properties in HBE cells

**Figure 5:**
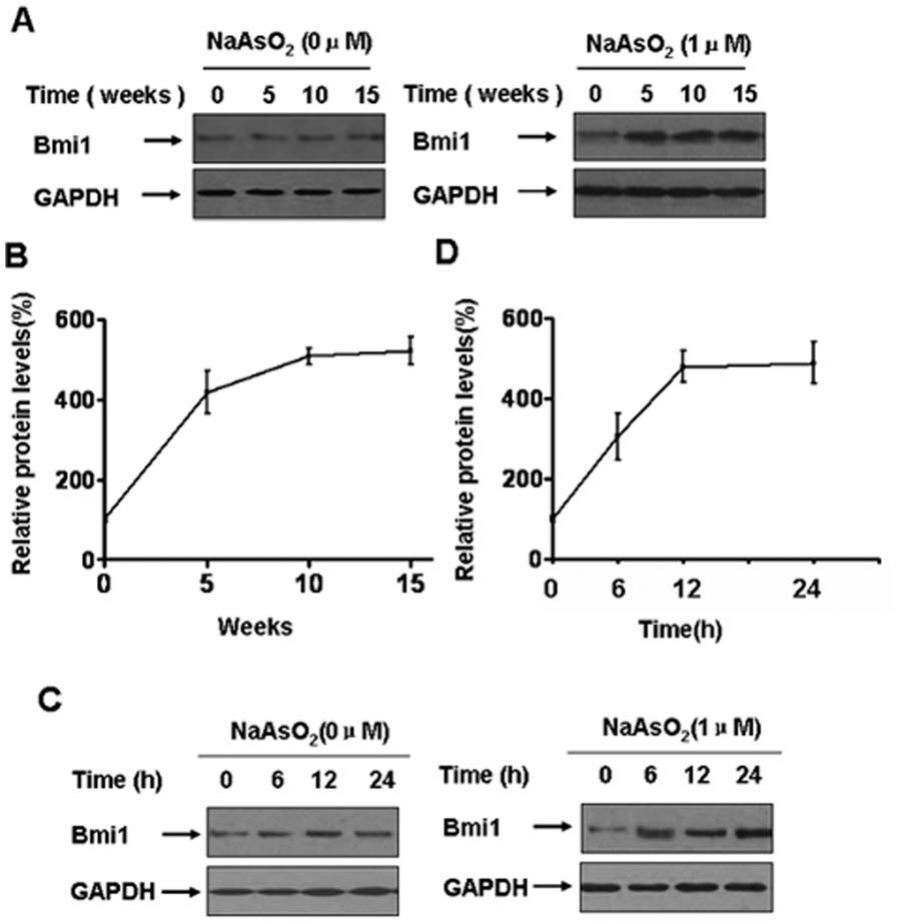
Bmi1 is involved in arsenite-induced acquisition of stem cell-like properties in HBE cells. Densities of bands were quantified by Eagle Eye II software. GAPDH levels, measured in parallel, served as controls. HBE cells were exposed to 0.0 or 1.0 µM arsenite for 5, 10, or 15 weeks. Western blots (**A**) and relative protein levels (**B**, means ± SD, n = 3) of Bmi1 were determined in control and treated HBE cells at the indicated times. Western blots (**C**) were performed and relative protein levels (**D**, means ± SD, n = 3) of Bmi1 were measured after HBE cells were exposed to 0.0 or 1.0 µM arsenite for 0, 6, 12, or 24 h.

Of the self-renewal genes necessary for arsenite-mediated maintenance of stem cells, Bmi1 has been reported to be causal for the transformation of cells [25]. However, the function of Bmi1 in arsenite-induced transformation remains unknown. Based on our results and others, the function of Bmi1 in arsenite-treated cells was investigated. In HBE cells chronically exposed to arsenite, the levels of Bmi1 increased with increased weeks of exposure (Figures 5A and 5B). Moreover, the levels of Bmi1 increased in cells exposed to arsenite for 6, 12, or 24 h (Figures 5C and 5D).

### In arsenite-induced EMT, HIF-2α regulates the levels of Twist1 and Bmi1 and the stem-like properties of HBE cells

**Figure 6:**
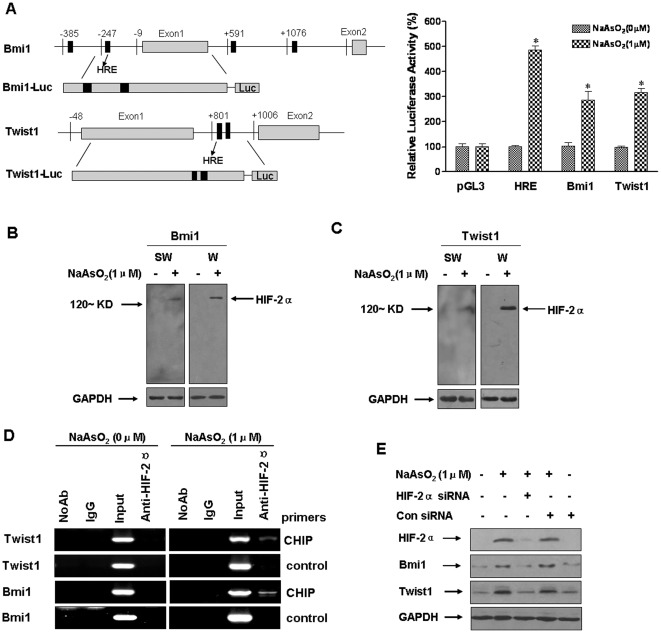
HIF-2α regulates Bmi1 and Twist1 in arsenite-induced EMT and acquisition of stem cell-like properties. (**A**) Schematic representation of the regulatory region of the *Bmi1* and *Twist1*genes and reporter constructs used in transfection assays. The luciferase reporter constructs were made with HRE, the *Bmi1* regulatory region (Bmi1–Luc), or the *Twist1* regulatory region (Twist1-Luc). HBE cells were co-transfected with a promoter construct and the indicated vector in control and arsenite-treated HBE cells for 24 h. Luciferase activity was measured and normalized according to Renilla luciferase activity (means ± SD, n = 3); ^*^ *P*<0.05 different from control cells. HBE cells were incubated with 0.0 or 1.0 µM arsenite for 24 h. (**B**) A band of about 120-kDa interacts with the probe of Bmi1 (SW). The HIF-2α antibody was incubated with the same membrane, and a 120-kDa band was identified (W). (**C**) The band of about 120-kDa interacts with the probe of Twist1 (SW). The HIF-2α antibody was incubated with the same membrane, and a 120-kDa band was also identified (W). (**D**) After the chromatin was immunoprecipitated using antibodies against HIF-2α, the binding of HIF-2α to promoters of Bmi1 and Twist1 were measured by a ChIP assay. (**E**) After HBE cells were exposed to 20 nM of control siRNA or to 10 nM HIF-2α siRNA for 24 h, they were incubated with 0.0 or 1.0 µM arsenite for 24 h. Western blots of HIF-2α, Twist1, and Bmi1 were performed.

In stem cells, HIF proteins maintain an undifferentiated state and are essential regulators for EMT [26]. The present results show that arsenite up-regulates the stabilization and transactivation of HIF-2α by inhibiting the ubiquitin-proteasome pathway under normoxic conditions (Figure S2). As determined by reporter assays, the HIF-2α-dependent transcriptional activity in HBE cells is activated by arsenite, and Twist1-Luc and Bmi1-Luc respond to arsenite stimulation (Figure 6A), suggesting that HIF-2α regulates Twist1 and Bmi1 expression by directly binding to its promoter. Since the DNA sequence (GGGCGGCGCGTGTGGCGCTG) of the *Bmi1,* and (GTGTGTGCGCGTGAGTGTGCGTGACAGGAG) of the *Twist1* promoters contain an hypoxia-responsive element [HRE, (A/G)CGTG], Southwestern and Western blots were used to determine if HIF-2α induces Bmi1 and Twist1 directly. The results revealed a band with a molecular weight of ∼120 kDa. The protein was identified as HIF-2α by incubation of the membrane obtained by Southwestern analysis with anti-HIF-2α antibody (Figure 6B and 6C). It is possible that the increases in Bmi1 and Twist1 were induced by activation of HIF-2α. To further examine the binding of HIF-2α to the Bmi1 and Twist1 promoter, a chromatin immunoprecipitation (ChIP) assay was performed. Upon arsenite exposure, HIF-2α bound to the Bmi1 and Twist1 gene promoters. In contrast, IgG did not associate with the Bmi1and Twist1 promoters at a detectable level (Figure 6D). HIF-2α knockdown abolished the increases of Twist1 and Bmi1 expression induced by arsenite (Figure 6E), suggesting that HIF-2α directly regulates Twist1 and Bmi1 in arsenite-induced EMT and the stem-like properties of HBE cells.

## Discussion

Inorganic arsenic is a widely distributed, naturally occurring environmental contaminant affecting tens of millions of people worldwide [27]. Chronic exposure to arsenic causes carcinogenesis of lung, skin, and bladder [28], [29]. Although there is evidence for the lung carcinogenicity of inorganic arsenic compounds in humans, the molecular mechanisms remain incompletely defined.

EMT refers to a program during normal embryonic development featuring a loss of epithelial properties, such as cell adhesion and expression of the epithelial marker, E-cadherin, and acquisition of mesenchymal properties, such as increased cell motility and expression of the mesenchymal marker, vimentin [1]. EMT, which is viewed as an important step in tumor invasion and metastasis [15], has not, however, been regarded as involved in malignant transformation of normal cells, that is, the initiation of tumorigenesis. The exposure of cells to arsenite or tobacco carcinogens induces EMT during transformation and tumor formation [3], [14], suggesting that the regulation of EMT morphology, induction of a stem cell-like phenotype, and transformation are distinct events in response to carcinogen exposure. In the present study, chronic arsenite exposure induced the EMT in HBE cells. Thus, arsenite-induced EMT of HBE cells is associated with transformation.

The process of EMT is controlled by transcriptional factors, including the zinc finger proteins, Snail, Slug, ZEB1, and ZEB2/SIP1, as well as the basic helix-loop-helix factor, Twist1. These transcriptional factors have been implicated in the transcriptional repression of E-cadherin in the induction of EMT [16]. In cells chronically exposed to arsenite, the EMT regulators, ZEB1 and ZEB2, are repressors of expression of E-cadherin, thus resulting in EMT [14]. Other regulators, however, have not been considered to be involved in arsenite induced-EMT. In the present study, which involved comparison of ZEB1, ZEB2, Slug, Snail, and Twist1 in control and arsenite-exposed cells, there was differential induction of these EMT regulators. Twist1 was induced by arsenite exposure, but Snail and Slug were not appreciably altered. ZEB1 and ZEB2 expressions were detected only in arsenite chronic-exposed cells, but there were no changes in cells acutely-exposed to arsenite. This was also observed by Wang et al. [14]. Therefore, we concluded that Twist1 is involved in the process of arsenite induced-EMT, but ZEB1and ZEB2 are involved only in the later period of arsenite-induced EMT. These results suggest that, along with ZEB1 and ZEB2, Twist1 is a novel transcription factor that is involved in arsenite-induced EMT.

The morphologic heterogeneity of lung tumors suggests that EMT is a process that drives differentiation and dedifferentiation in early tumor development [30]. The induction of human mammary epithelial cells into the EMT phenotype was concomitant with the acquisition of the CD44^high^/CD24^low^ expression pattern and increased mammosphere-forming and tumor-initiating capacity [6], [31]. A recent investigation found that the induction of EMT resulted in an increased population of CD44^high^/CD24^low^ cells and drug resistance associated with CSC signatures [32]. These reports suggest that cells with an EMT phenotype, induced by different factors, are rich sources for CSCs.

Stem cells are probably a key target in arsenic carcinogenesis. Arsenic induces malignant transformation of stem cells *in vitro* [11] and causes an overproduction of CSCs that is associated with an acquired malignant phenotype or cancer production [10], [13]. In the present study, cells exposed to arsenite for induction of EMT exhibited enhanced capacity for forming mammospheres and CSCs, compared to control cells. Expressions of CD133 and CD44 were substantially increased in the arsenite-induced EMT.

SPs of cells, which are enriched in primitive and undifferentiated CSCs, are long-lived, self-renewing, and highly proliferative [33]. In the present investigation, the percentage of SP cells increased in the arsenite-induced EMT of HBE cells. These results indicate that arsenite converts normal cells to CSCs.

Stem cells and CSCs share a variety of properties, including self-renewal, although it is typically dysregulated in CSCs [34]. In embryonic stem cells, embryonic epiblasts, and primordial germ cells, Oct4 is essential for maintaining an undifferentiated state, [22]. In several types of stem cells, Bmi-1 is implicated in the modulation of self-renewal [34], [35]. For several cell types, the Notch pathway controls the fate of stem cells [34], and, for humans, ALDH1 is a marker of normal and malignant mammary stem cells [23]. Sox2 is a transcription factor essential for the pluripotent and self-renewing phenotypes of CSCs [24]. The expressions of *Oct4*, *Bmi1*, and *ALDH1* are involved in maintaining cancer stem-like cells in lung cancer. They also have a role in epithelial tumorigenesis and provide a molecular link between putative lung stem cells and lung tumorigenesis [36], [37], [38], [39]. The present study shows that there is up-regulation of expressions of Oct4, Bmi1 and ALDH1 in chronic exposure to arsenite. This observation is consistent with the concept that the self-renewal genes, *Oct4*, *Bmi1*, and *ALDH1* are necessary for arsenite-mediated maintenance of cancer stem-like cells.

In the present study, we found that arsenite produces CSCs during malignant transformation of HBE cells, and, during this process, the HBE cells acquire a fibroblast-like mesenchymal appearance consistent with EMT. However, the process of EMT is unlikely to be involved in acquisition of stem cell-like features. Arsenite-induced EMT and the acquisition of stem cell-like features may be associated with arsenite-induced malignant transformation. Further studies are needed to elucidate the mechanisms by which the stem-like cells are selected due to the treatment or induction of EMT.

Hypoxia-inducible factors (HIFs) maintain an undifferentiated state in stem cells [40]. In particular, *Oct4* is a target gene for HIF-2α, indicating that HIF-2α regulates stem cell function and/or differentiation through activation of *Oct4* [41]. Additionally, HIF-2α could induce EMT through transcription of EMT regulators [42]. Hence, based on these findings, HIF-2α may be necessary for arsenite-mediated induction of EMT and for maintenance of cancer stem-like cells. Apparently, HIF-2α could regulate Twist1 in cells undergoing an EMT, and Bmi1 is essential for Twist1-induced EMT and tumor-initiating capacity [43], we found that HIF-2α regulates Bmi1 and Twist1 transcription by directly binding to their promoters under arsenite exposure.

The present study focused on the induction and function of Bmi1 and Twist1 in cells chronically exposed to arsenite. Nevertheless, other self-renewal genes, such as *ALDH1*, may be necessary for arsenite-mediated maintenance of cancer stem-like cells. Thus, further study is required to determine if higher expression and function this gene is necessary for arsenite-mediated maintenance of cancer stem-like cells.

We first reported that, during arsenite exposure, HIF-2α directly induces Bmi1 expression through binding to HREs in their promotor region, not by mediation of twist1 [43]. These results provide support for an essential function of HIF-2α in arsenite-mediated induction of EMT and in maintenance of cancer stem-like cells.

In conclusion, this investigation expands our understanding of the carcinogenic potential of arsenite by indicating that it can targets CSCs for carcinogenic transformation. Arsenite-induced oncogenic changes associated with HIF-2α are induction of EMT and the development of a cancer stem cell-like phenotype during malignant transformation. These observations contribute to a better understanding of the processes involved in arsenite-induced carcinogenesis.

## Materials and Methods

### Cell culture and reagents

HBE cells, a SV40-transformed normal human bronchial epithelial cell line, are nontumorigenic and retain features of human bronchial epithelial cells; they are useful for studies of multistage bronchial epithelial carcinogenesis [44]. This cell line was obtained from the Shanghai Institute of Cell Biology, Chinese Academy of Sciences (Shanghai, China). HBE cells were maintained in 5% CO_2_ at 37°C in Minimum Essential Medium Eagle's medium (MEM), supplemented with 10% fetal bovine serum (FBS, Life Technologies/Gibco, Grand Island, NY), 100 U/ml penicillin, and 100 µg/ml streptomycin (Life Technologies/Gibco, Gaithersburg, MD). For chronic exposure, 1×10^6^ cells were seeded into 10-cm (diameter) dishes for 24 h and maintained in 0.0 or 1.0 µM sodium arsenite (NaAsO_2_, Sigma, St. Louis, MO, purity: 99.0%) for 48–72 h per passage. This process was continued for about 15 weeks. All other reagents used were of analytical grade or highest grade available.

### Western blots

Total cell lysates were separated by sodium dodecyl sulfate-polyacrylamide gel electrophoresis (SDS-PAGE) and were transferred to polyvinylidene fluoride membranes (Millipore, Billerica, MA, USA); the immune complexes were detected by enhanced chemiluminescence (Cell Signaling Technology, Beverly, MA). Antibodies used were: Twist1, Bmi1 and glyceraldehyde 3-phosphate dehydrogenase (GAPDH, Sigma); hypoxia-inducible factor-2α (HIF-2α, Novus, Littleton, CO); E-cadherin, N-cadherin, vimentin, Snail, Slug, ZEB1, and ZEB2 (Cell Signaling Technology). Blots were quantitated by densitometry and normalized using GAPDH to correct for differences in protein loading. For densitometric analyses, protein bands on the blot were measured by use of Eagle Eye II software.

### RNA interference

The transfections were performed on HBE cells with the N-TER™ Nanoparticle siRNA Transfection System (Sigma) following the manufacturer's protocol. Briefly, 5×10^5^ cells were seeded into each well of 6-well plates, 18–24 h prior to transfection. The siRNA nanoparticle formation solution (NFS) was prepared by adding target gene siRNA dilutions to N-TER peptide dilutions and incubated at room temperature for 30 min. NFS transfection medium (2 mL) containing target gene siRNA was transferred to each well of the culture plates, and, after for 24 h, cells were treated and harvested for analysis. Control siRNA was purchased from Santa Cruz Biotechnology (Santa Cruz, CA). HIF-2α siRNA was purchased form Abnova Corporation (Abnova, CA).

### Quantitative real-time PCR

Total RNA was extracted, and RT-PCR was performed as described previously [45]. Total RNA (2 µg) was transcribed into cDNA by use of AMV Reverse Transcriptase (Promega, Madison, Wisconsin, USA). Primers used are listed (Table S1). Quantitative real-time PCR was performed using the Applied Biosystems 7300HT machine and Maxima™ SYBR Green/ROX qPCR Master Mix (Fermentas, USA). The PCR reaction was evaluated by melting curve analysis and by checking the PCR products on 2% w/v agarose gels. *GAPDH* was amplified to ensure cDNA integrity and to normalize expression.

### Southwestern assays

Southwestern analyses were performed as described previously [46]. Briefly, nuclear extracts (80 µg) of HBE cells were separated by SDS-PAGE and transferred to nitrocellulose membranes (Millipore). After transferring, the filters were hybridized for 2 h at 20°C with binding buffer containing 40 ng of the biotin-labeled probe for the promoter of Bmi1: GGGCGGCGCGTGTGGCGCTG, and the promoter of Twist1: GTGTGTGCGCGTGAGTGTGCGTGACAGGAG. The filters were then washed in binding buffer at 20°C for 20 min. The positions of the biotin end-labeled oligonucleotides were detected by a chemiluminescent reaction according to the manufacturer's instructions (Pierce, Rockford, IL) and visualized by autoradiography.

### Transient Transfection and Luciferase Activity Assay

The pGL3-HRE-Luc construct, pGL3-Bmi1-Luc construct, and the pGL3-Twist1-Luc construct were purchased from Shuntian Biology (Shanghai). The plasmid phRL-tk (used as internal control for transfection efficiency and cytotoxicity of test chemicals) containing the Renilla luciferase gene was purchased from Promega (Madison, WI, USA). HBE cells (2×10^6^) were plated in 100-mm cell culture dishes. The cells proliferated to 60 to 80% confluence after 24 h of culture. Then, 3 µg of DNA of the reporter constructs was transfected into cells using the Lipofectamine 2000 reagent (Invitrogen, Carlsbad, CA) according to the manufacturer's protocol. After an incubation period of 12 h, the transfection medium was replaced. The cells were harvested after being exposed to arsenite for 24 h. After three rinses with PBS (pH 7.4), the cells were lysed with 1×passive lysis buffer (Promega). The cell lysates were analyzed immediately with a 96-well plate luminometer (Berthold Detection System, Pforzheim, Germany). The amounts of luciferase and Renilla luciferase were measured with the Dual-Luciferase Reporter Assay System Kit (Promega) following the manufacturer's instructions. The values of luciferase activity for each lysate were normalized to the Renilla luciferase activity. The relative transcriptional activity was converted into fold induction above the vehicle control value.

### Chromatin immunoprecipitation (ChIP)

ChIP was performed as described Lee at el. [47]. Cells (1×10^7^) were treated with or without arsenite for 24 h and cross-linked in 1% formaldehyde for 10 min. After cell lysis, the chromatin was fragmented to an average size of 500 bp and enriched with magnetic Dynal bead (Invitrogen)-coupled antibody against HIF-2α, with no antibody, or with isotype IgG at 4°C overnight. The cross-links for the enriched and the input DNA were then reversed, and the DNA was cleaned by RNase A (0.2 mg/ml) and proteinase K (2 µg/ml) before phenol/chloroform-purification. The specific sequences from immunoprecipitated and input DNA were determined by PCR primers for Bmi1 and Twist1 promoters upstream regions, and their respective control primers not containing HRE binding elements: Bmi1 promoter forward, 5-*GGCCTCGCCGCCGGCGCG*-3, and reverse, 5- *CTCCCCTCGTGCACTGGGCG*-3, the amplicon size was 189 bp; Bmi1 control promoter forward, 5- *CGCCGCGGCCTCGGACC* -3, and reverse, 5- *GCACGCCCCGGCCTCG* -3, the amplicon size was 144 bp; Twist1 promoter forward, 5- *TTCCGGCCAGACTGGGGC* -3, and reverse, 5- *CTGGCAAAACAGTCGCGG* -3, the amplicon size was 141 bp; Twist1 control promoter forward, 5- *TCGTCGTCGCCGCCGCCCTC* -3, and reverse, 5- *GGGTGCGACGGGAGCCTG* -3, the amplicon size was 147 bp.

### Immunostaining

Immunostaining analyses were performed as described previously [46]. Briefly, HBE cells were stained with rabbit E-cadherin and vimentin antibody at 4°C overnight and then incubated with Cy3-conjugated goat-anti-rabbit secondary antibody (Millipore, Billerica, MA, USA) for 1 h. To stain the nuclei, 4′, 6-diamidino-2-phenylindole (DAPI, Sigma) was added for 10 min, and the cells were observed under a fluorescence microscope (Olympus, Shinjuku-ku, Tokyo, Japan). The fluorescence intensities were measured with a multimode microplate reader (TECAN, Trading, AG, Switzerland), and images were analyzed with an Image-Pro Plus 6.0 (Olympus).

### Analysis of side populations (SPs)

The HBE cells were removed from the culture dish with trypsin and EDTA, washed, suspended at 10^6^ cells/ml in DMEM/F-12 (Dulbecco's Modified Eagle Medium: Nutrient Mixture F-12; Gibco-BRL) containing 5% FBS (staining medium), and incubated in a 1.5-ml Eppendorf tube at 37°C for 10 min. The cells were then labeled in the same medium at 37°C for 90 min with 5.0 µg/ml Hoechst 33342 (Sigma) dye, either alone or in combination with 50 µM verapamil (Sigma), an inhibitor of ATP-binding cassette (ABC) transporters. The cells were counterstained with 1 mg/ml of propidium iodide (Sigma) to label dead cells. Then, 10^5^ cells were passed through a FACSVantage fluorescence-activated cell sorter (Becton Dickinson, East Rutherford, NJ, USA) and subjected to dual-wavelength analysis (blue, 424–444 nm; red, 675 nm) after excitation with 350 nm UV light [43].

### Spheroid formation

In nonadherent dishes (Costar, US), **HBE** cells (1×10^4^) were suspended in defined, serum-free medium composed of DMEM/F-12, 10 ng/ml human recombinant basic fibroblast growth factor (bFGF, R&D Systems) and 10 ng/ml epidermal growth factor (EGF, R&D Systems). The spheroids were resuspended to form secondary spheroids. The medium was changed daily along with growth factor supplementation. For formation of secondary spheres, dissociated cells of primary spheres were washed at least three times and then plated on nonadherent plates at the desired cell densities for an additional 10 days [43].

### Statistical analysis

A one-way analysis of variance (ANOVA) was used to assess differences among groups. Statistical significance was determined by the Student's test. *P* values <0.05 were considered statistically significant. Derived values are presented as the means ± SD.

## Supporting Information

Experimental Procedures S1 **Anchorage-independent growth.** The method is used in Figure S1.
(DOC)

Experimental Procedures S2 **Tumorigenicity in intact animals.** The method is used in Figure S1.
(DOC)

Experimental Procedures S3 **Co-immunoprecipitation.** The method is used in Figure S2.
(DOC)

Figure S1 **Neoplastic transformation of HBE cells induced by 1.0 µM arsenite.** Abbreviations: HBE, passage control HBE cells; T-HBE, arsenite-transformed HBE cells; A549, A549 carcinoma cells. HBE cells were exposed to 0.0 or 1.0 µM sodium arsenite for about 15 weeks (30 passages). A549 cells served as a positive control. Cell colonies (**A**) and their number (**B**, means ± SD, n = 3) in soft agar; bars = 100 µm (Experimental Procedures S1). Cells were injected into nude/BalbC mice. At 4 weeks after inoculation of the cells. (**C**) tumors that formed from the transformed cells and A549 cells were examined and (**D**) their volumes were measured (means ± SD, n = 6). ^**^ *P*<0.01 difference from medium control cells (Experimental Procedures S2). (**E**) Histological examination of the implanted sites of the mice shown in (C) by haematoxylin and eosin (H&E) stains. Tumors induced by arsenite-transformed cells were composed of typical undifferentiated squamous epithelium and scar-like tissues; bars = 250 µm.
(TIF)

Figure S2 **Effects of arsenite on the degradation of HIF-2α in HBE cells.** Densities of bands were quantified by Eagle Eye II software. GAPDH levels, measured in parallel, served as controls. HBE cells were exposed to 0.0 and 1.0 µM arsenite for 0, 1, 3, 6, 9, 12, or 24 h, respectively. (**A**) Western blots of HIF-1α and HIF-2α were measured after HBE cells were treated by arsenite, or to 100 µM desferroxamine (DFX) for 12 h. The mRNA level of *HIF-2α* were determined by RT-PCR (**B**) and by quantitative PCR (**C**, means ± SD, n = 3). After HBE cells were exposed to 1.0 µM arsenite for 24 h, then such cells were treated with protein synthesis inhibitor Cycloheximide (CHX, 10 µg/ml) in the absence or presence of arsenite for the times indicated. Western blot (**D**) and the levels of protein remaining (**E**, means ± SD, n = 3) of HIF-2α were investigated. *P<0.05 and **P<0.01 difference from cells treated with CHX and arsenite. After HBE cells were treated with 1.0 µM arsenite, 10 µM proteasome inhibitor MG132, or a combination of these two reagents for 12 h, the levels of HIF-2α and modfied-HIF-2α, were analysed by Western blot analyses (**F**). Cells were treated as described in (F), such cells were subjected to co-immunoprecipitation with HIF-2α (IP) and ubiquitin (IB) antibodies (Experimental Procedures S3). Levels of HIF-2α and ubiquitinated-HIF-2α were determined by Western blot (**G**).
(TIF)

Table S1 **Primers Sequences Used.** Primers sequences used are listed in Table S1.
(DOC)
