# Supplementary material for: EMT and Stem Cell-Like Properties Associated with HIF-2α Are Involved in Arsenite-Induced Transformation of Human Bronchial Epithelial Cells
Source: PLoS One. 2012 May 25;7(5):e37765. doi: 10.1371/journal.pone.0037765 (PMC3360629; doi:10.1371/journal.pone.0037765)
Supplement: Experimental Procedures S1 — Anchorage-independent growth. The method is used in Figure S1. (DOC) [file pone.0037765.s001.doc]

**Experimental Procedures S1. Anchorage-independent growth.** Soft agar dishes were prepared with under-layers of 0.70% agarose in MEM medium supplemented with 10% FBS. To test for capacity for soft-agar growth, treated HBE cells were plated in triplicate at a density of 1×10^4^ in 2 mL of 0.35% agarose over the agar base. Cultures were fed every three days. After for 14 days, colonies with >30 cells were counted.
