# Supplementary material for: EMT and Stem Cell-Like Properties Associated with HIF-2α Are Involved in Arsenite-Induced Transformation of Human Bronchial Epithelial Cells
Source: PLoS One. 2012 May 25;7(5):e37765. doi: 10.1371/journal.pone.0037765 (PMC3360629; doi:10.1371/journal.pone.0037765)
Supplement: Experimental Procedures S2 — Tumorigenicity in intact animals. The method is used in Figure S1. (DOC) [file pone.0037765.s002.doc]

**Experimental Procedures S2. Tumorigenicity in intact animals.**

According to a protocol approved by the Nanjing Medical University Institutional Animal Care and Use Committee, animals were treated humanely and with regard for alleviation of suffering. Briefly, 1×10^7^ cells were injected subcutaneously into the right armpit of nude mice (BalbC mice, Shanghai Lab. Animal Research Center, China). There were 6 mice per group. Tumor incidence and size were monitored once per week. Tumors exceeding 3 mm diameter were recorded as positive. Animals were euthanized and tumors were castrated when one of the animals body weight dropped by more than 20% or tumor diameter exceeded 15 mm. Four weeks later, the tumor tissues were removed, fixed with 4% formalin, embedded in paraffin, sectioned, stained with hematoxylin and eosin, and analyzed by light microscopy.
