# Supplementary material for: EMT and Stem Cell-Like Properties Associated with HIF-2α Are Involved in Arsenite-Induced Transformation of Human Bronchial Epithelial Cells
Source: PLoS One. 2012 May 25;7(5):e37765. doi: 10.1371/journal.pone.0037765 (PMC3360629; doi:10.1371/journal.pone.0037765)
Supplement: Experimental Procedures S3 — Co-immunoprecipitation. The method is used in Figure S2. (DOC) [file pone.0037765.s003.doc]

**Experimental Procedures S3. Co-immunoprecipitation**.

Cells were extracted for 30 min with lysis buffer. After centrifugation of the preparations, the supernatants were incubated with HIF-2α antibody and subsequently with A+G Sepharose beads (Sigma) at 4 °C overnight. The pellets were washed three times, re-suspended in the SDS sample buffer, and boiled to remove protein from the beads. The immunoprecipitates were analyzed by Western blots with ubiquitin, HIF-2α antibodies, respectively.
