# Supplementary figures and images for: EMT and Stem Cell-Like Properties Associated with HIF-2α Are Involved in Arsenite-Induced Transformation of Human Bronchial Epithelial Cells
Source: PLoS One. 2012 May 25;7(5):e37765. doi: 10.1371/journal.pone.0037765 (PMC3360629; doi:10.1371/journal.pone.0037765)

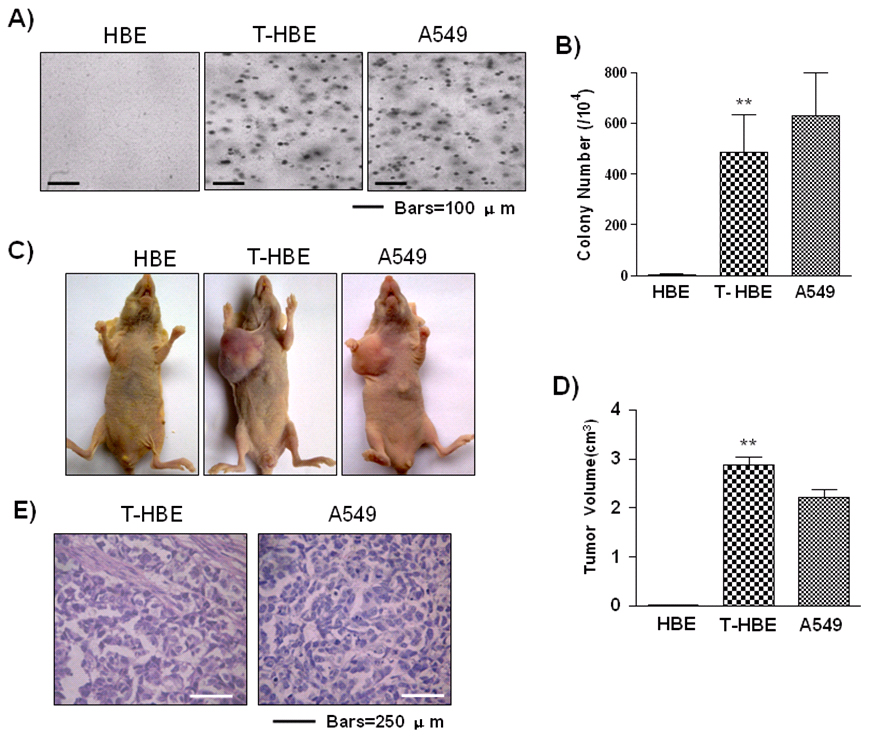

Supplement: Figure S1 — Neoplastic transformation of HBE cells induced by 1.0 µM arsenite. Abbreviations: HBE, passage control HBE cells; T-HBE, arsenite-transformed HBE cells; A549, A549 carcinoma cells. HBE cells were exposed to 0.0 or 1.0 µM sodium arsenite for about 15 weeks (30 passages). A549 cells served as a positive control. Cell colonies (A) and their number (B, means ± SD, n = 3) in soft agar; bars = 100 µm (Experimental Procedures S1). Cells were injected into nude/BalbC mice. At 4 weeks after inoculation of the cells. (C) tumors that formed from the transformed cells and A549 cells were examined and (D) their volumes were measured (means ± SD, n = 6). ** P<0.01 difference from medium control cells (Experimental Procedures S2). (E) Histological examination of the implanted sites of the mice shown in (C) by haematoxylin and eosin (H&E) stains. Tumors induced by arsenite-transformed cells were composed of typical undifferentiated squamous epithelium and scar-like tissues; bars = 250 µm. (TIF) [file pone.0037765.s004.tif]

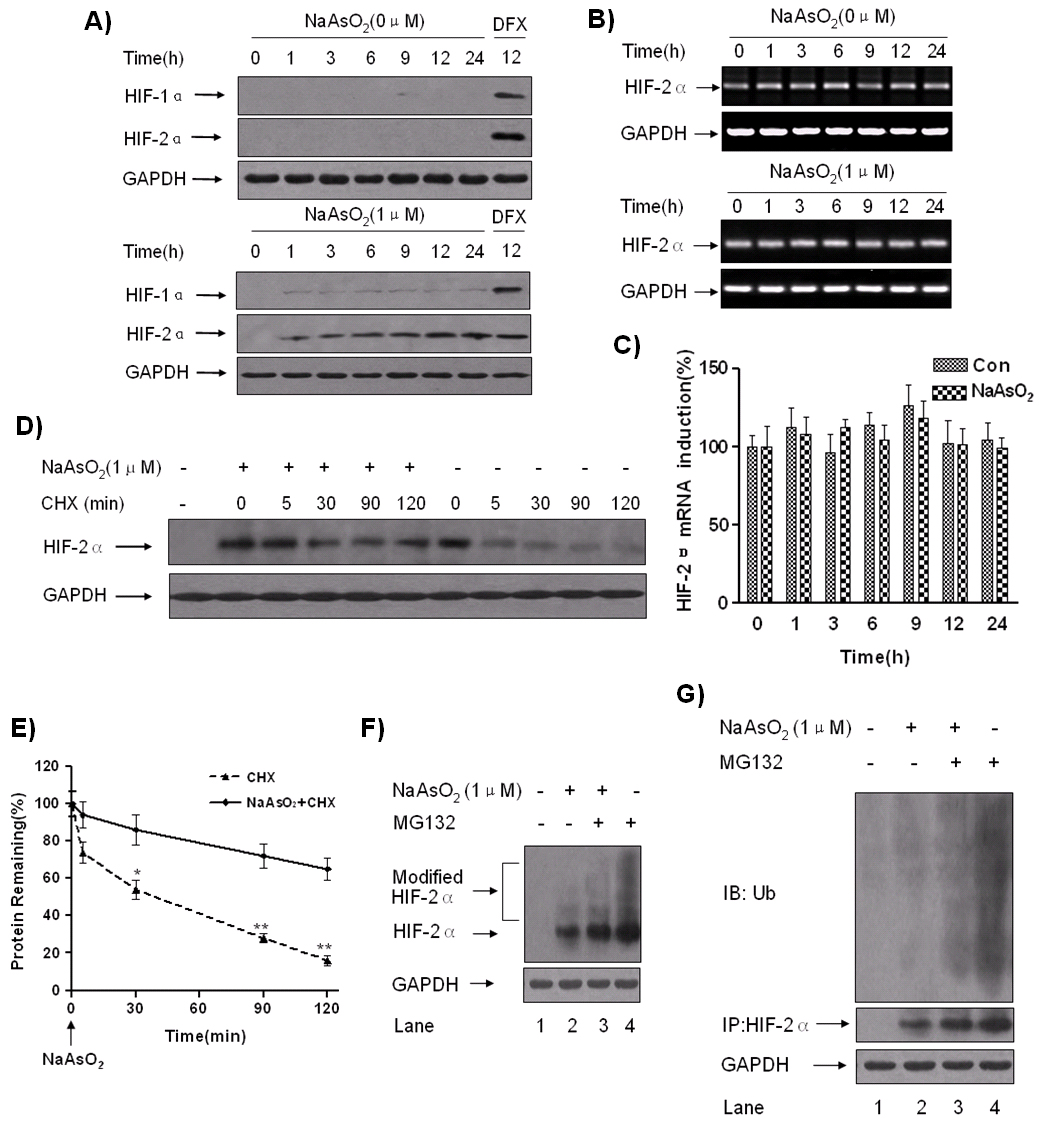

Supplement: Figure S2 — Effects of arsenite on the degradation of HIF-2α in HBE cells. Densities of bands were quantified by Eagle Eye II software. GAPDH levels, measured in parallel, served as controls. HBE cells were exposed to 0.0 and 1.0 µM arsenite for 0, 1, 3, 6, 9, 12, or 24 h, respectively. (A) Western blots of HIF-1α and HIF-2α were measured after HBE cells were treated by arsenite, or to 100 µM desferroxamine (DFX) for 12 h. The mRNA level of HIF-2α were determined by RT-PCR (B) and by quantitative PCR (C, means ± SD, n = 3). After HBE cells were exposed to 1.0 µM arsenite for 24 h, then such cells were treated with protein synthesis inhibitor Cycloheximide (CHX, 10 µg/ml) in the absence or presence of arsenite for the times indicated. Western blot (D) and the levels of protein remaining (E, means ± SD, n = 3) of HIF-2α were investigated. *P<0.05 and **P<0.01 difference from cells treated with CHX and arsenite. After HBE cells were treated with 1.0 µM arsenite, 10 µM proteasome inhibitor MG132, or a combination of these two reagents for 12 h, the levels of HIF-2α and modfied-HIF-2α, were analysed by Western blot analyses (F). Cells were treated as described in (F), such cells were subjected to co-immunoprecipitation with HIF-2α (IP) and ubiquitin (IB) antibodies (Experimental Procedures S3). Levels of HIF-2α and ubiquitinated-HIF-2α were determined by Western blot (G). (TIF) [file pone.0037765.s005.tif]
