# Supplementary material for: EMT and Stem Cell-Like Properties Associated with HIF-2α Are Involved in Arsenite-Induced Transformation of Human Bronchial Epithelial Cells
Source: PLoS One. 2012 May 25;7(5):e37765. doi: 10.1371/journal.pone.0037765 (PMC3360629; doi:10.1371/journal.pone.0037765)
Supplement: Table S1 — Primers Sequences Used. Primers sequences used are listed in Table S1. (DOC) [file pone.0037765.s006.doc]

| *CD44* | 5'- TGAGCATCGGATTTGAGAC -3' |
| --- | --- |
|  | 5'- CATACTGGGAGGTGTTGGA -3' |
| *CD133* | 5'- TCGGAAACTGGCAGATAGC -3' |
|  | 5'- GAACGCCTTGTCCTTGGT -3' |
| *Bmi1* | 5'- CTGATGACCCATTTACTGA -3' |
|  | 5'- CTCCACCTCTTCTTGTTTG -3' |
| *ALDH1* | 5'- TGTCCAAGTCGGCATCAG -3' |
|  | 5'- GGCAGCCATTTCTTCTCA -3' |
| *Oct4* | 5'- GCTTCCTCCACCCACTTCT -3' |
|  | 5'- GTATTCAGCCAAACGACCAT-3' |
| *SOX2* | 5'- CTCCCATTTCCCTCGTTT -3' |
|  | 5'- GGTTACCTCTTCCTCCCACT -3' |
| *Notch1* | 5'- CCGCCTTTGTGCTTCTGTTC -3' |
|  | 5'- CGCCGCTTCTTCTTGCTG -3' |
| *HIF-2α* | 5'- CACCAAGGGTCAGGTAGTAAG -3' |
|  | 5'- GGTTGCGAGGGTTGTAGAT -3' |
| *E-cadherin* | 5'- TGCTCACATTTCCCAACTC -3' |
|  | 5'- TCTGTCACCTTCAGCCATC -3' |
| *N-cadherin* | 5'-GGGTAATCCTCCCAAATC-3' |
|  | 5'-TTCTCCTCCACCTTCTTCA -3' |
| *Vimentin* | 5'- CCAGGCAAAGCAGGAGTC -3' |
|  | 5'- GGGTATCAACCAGAGGGAGT -3' |
| *GAPDH* | 5'- GACCTGACCTGCCGTCTA -3' |
|  | 5'- GGAGTGGGTGTCGCTGT-3' |
